# Association between patient-provider communication and withholding information due to privacy concerns among women in the United States: an analysis of the 2011 to 2018 Health Information National Trends Survey

**DOI:** 10.1186/s12913-023-10112-7

**Published:** 2023-10-25

**Authors:** Kobi V. Ajayi, Samson Olowolaju, Obasanjo Afolabi Bolarinwa, Henry Onyeka

**Affiliations:** 1 Department of Health Behavior, School of Public Health, Texas A&M University College Station, College Station, TX USA; 2 Department of Demography, College for Health, Community and Policy, University of Texas, San Antonio, TX USA; 3 Department of Public Health, York St. John University, London, UK; 4 Department of Demography and Population Studies, University of Witwatersrand, Johannesburg, South Africa; 5 Department of Psychiatry, Harvard Medical School, Boston, MA USA; 6 Department of Psychiatry, Massachusetts General/Mclean Hospital, Boston, MA USA

**Keywords:** Women’s health, Patient-provider communication, Electronic medical records, Digital technology, United States

## Abstract

**Background:**

Electronic medical record software is common in healthcare settings. However, data privacy and security challenges persist and may impede patients’ willingness to disclose health information to their clinicians. Positive patient-provider communication may foster patient trust and subsequently reduce information nondisclosure. This study sought to characterize information-withholding behaviors among women and evaluate the association between positive patient-provider communication and women’s health information-withholding behavior in the United States.

**Methods:**

Data were pooled from the 2011 to 2018 Health Information National Trends Survey. We used descriptive statistics, bivariate, and logistic regression analyses to investigate whether positive patient-provider communication significantly impacted health information-withholding behaviors. Data from 7,738 women were analyzed.

**Results:**

About 10.8% or 1 in 10 women endorsed withholding health information from their providers because of privacy or security concerns about their medical records. After adjusting for the covariates, higher positive patient-provider communication scores were associated with lower odds of withholding information from the provider because of privacy and security concerns (aOR 0.93; 95% CI = 0.90–0.95). Additionally, we found that age, race/ethnicity, educational status, psychological distress, and smoking status significantly predicted women’s willingness to disclose health information.

**Conclusions:**

Findings suggest that improving positive patient-provider communication quality may reduce women’s privacy and security concerns and encourage them to disclose sensitive medical information.

## Introduction

Electronic medical record (EMR) software is ubiquitous in health systems and organizations, with undeniable benefits in improving all aspects of the care continuum for patients, providers, and health systems alike [1–3]. Despite its advantages, concerns about the misuse of protected health information (PHI) persist with serious health and economic implications [4–6]. With the growing digitalization of medical care through technological advances and tools such as mobile devices, wearable electronic devices, and cloud-based services, the malicious use of PHI are real concerns for patients, dissuading them from disclosing information to their providers [6]. However, beyond the reasons above for withholding PHI, the impact of health policies [7, 8] on women’s health, compounded by the limitations in the Privacy Rule of the Health Insurance Portability and Accountability Act (HIPAA) and other information privacy laws, may limit women’s willingness to disclose crucial health information leading to suboptimal health care outcome [9].

Women’s health is broad and influenced by genetic, sociocultural, environmental, and sociopolitical factors [10]. Despite advancements and significant medical breakthroughs in the United States (US), women’s health is in crisis and remains an important public health issue. For example, American women have higher morbidity and mortality rates across several health indices than those from countries in the global north [11, 12]. Moreover, women’s bodies in the US are constantly under surveillance by the government and the public [7], defining where, when, and how they seek care, thus prompting a reliance on digitalized care because of the supposed “anonymity” they offer. Ironically, digital health exchange mediums (e.g., EMR, Internet searches, mHealth, mobile apps), which otherwise should protect women’s health privacy, are porous and fall short of this standard [13, 14]. For example, in 2019, Missouri kept track of women’s menstrual periods in clinics to monitor abortion attempts or offenders [15]. Similar surveillance policies on women’s reproductive health have been reported across the US [16].

Further, EMR, text messages, and web searches are used as evidence to substantiate criminal charges against women’s reproductive health choices [17]. As a result, women seeking certain reproductive health services might be unwilling to disclose critical health information, avoid necessary healthcare, or seek health from unlikely sources, with detrimental and devastating consequences. Such a precarious situation would exacerbate women’s health issues beyond reproductive health care in the US, considering that female patients tend to be more concerned about privacy and data security than men (84% vs. 71% and 80% vs. 66%) [18]. Although an individual may share their PHI when seeking health information on the web or app, individuals (not the website or app themselves) do not fall under the purview of HIPAA laws because they are not considered as covered entities. According to HIPAA [19], covered entities, such as providers in most cases, are permitted but not mandated to disclose PHI to patients or their representatives for treatments and payments purposes or other uses to facilitate patient care with their express consent. However, providers are mandated by law to disclose PHI in compliance with investigations or enforcement actions without the patient’s authorization. This clear distinction about what is considered as HIPAA vs. non-HIPAA disclosures is necessary for this discourse because even though surfing the internet are outside of HIPAA rules and stipulations, women are still criminalized for their choices outside of medical encounter [15–17] due to nuances and limitations in the interpretation of HIPAA.

Much theoretical evidence abounds in the discourse of nondisclosure of health information for privacy and security risks, clarifying the factors influencing consumers’ decisions to withhold information [20]. While most theories suggest that structural and individual-level determinants influence individuals’ willingness to disclose information, they are mainly conducted in the e-commerce milieu [20, 21]. These theories are applicable in understanding privacy concerns in the healthcare setting; after all, healthcare is transactional, albeit dynamic, with multiple and complex actors. Nonetheless, an ostensibly common thread found in the theories can be inferred. There is a trade-off on how individuals decide to disclose information based on perceived consequences (benefits or risks) associated with the information shared and the context in which they are shared, a central tenet of the privacy calculus theory and the theory of reasoned action/planned behavior [20, 21]. In light of the risk-benefit mindset, it is plausible that US women would consider the risk of breaches to their EMR as a substantiated reason to withhold vital health information from their providers.

Recently, there has been an increased call for strategies encouraging optimal healthcare delivery by ensuring data protection and privacy among women [13]. Quality and positive patient-provider communication (PPC) is a potentially modifiable factor that may enhance the communication dynamics during medical consultation, building trust and empathy to protect women’s health [22]. This is because PPC is embedded within the health communication and patient-centered care paradigm, underscoring the importance of quality communication in delivering quality and equitable health care [22]. It has been described as essential in enhancing health service utilization, adherence to treatment, and overall health outcomes [22, 23]. PPC is also associated with non-delay/avoidance of medical care among US women. For example, women with higher PPC scores were less likely to avoid a needed medical visit [24], even after adjusting for sociodemographic characteristics. However, one key question must be considered: to what extent do external factors (e.g., state and federal health policies or sociocultural context) affect patient-provider relationship quality in light of the everchanging health information exchange landscape in the US?

**Fig. 1:**
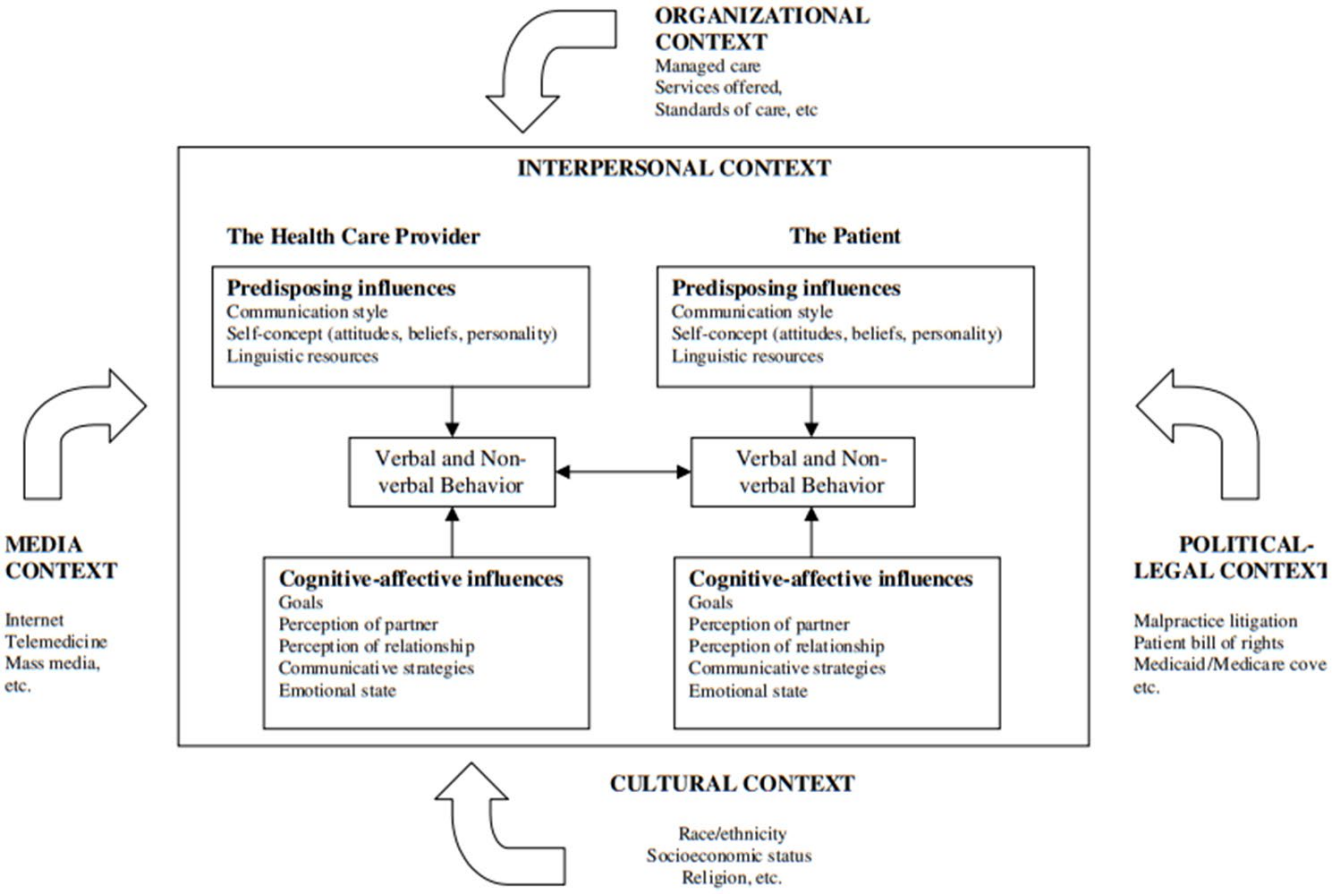
The ecological model of communication in medical encounters. *Reprinted with permission from Street Jr* [25]

To understand this question, the Ecological Model of Communication in Medical Encounters [25]( see Fig. 1) can be considered because it describes succinctly the multilevel mechanisms influencing PPC. The ecological perspective on health communication suggests that patient-provider interactions are affected by numerous proximal (e.g., communication style, linguistic style, verbal and non-verbal behaviors, and cognitive influences) and distal factors (e.g., interpersonal, organizational, media, political-legal, and cultural environments) wherein these interactions are situated. Indeed, in today’s clime, an ecological perspective on the patient-provider process may help build trust because it addresses these proximal and distal factors to mitigate women’s privacy concerns, thereby improving health outcomes.

While much is known about PPC across different domains of women’s health, very little is known about how or whether PPC influences their willingness to disclose health information for privacy reasons. Previous studies on PHI nondisclosures have measured several variables influencing information-withholding behaviors but not PPC [1, 26, 27]. Still, even though the literature suggests that women are more likely to withhold information from clinicians than their male counterparts, these studies are limited by sample size, have not used nationally representative data, or did not stratify by sex, which may not uncover important patterns relevant to women’s health: restricting the generalizability of these findings [1, 27–29]. Further, gender is a predisposing factor to effective PPC in that female patients tend to receive biased care and are often stereotyped, regardless of the gender of the providers [30]. This dilemma is even magnified for ethnic and minority women whose skin color further influences their care. Therefore, it is evident that more research is needed to evaluate if quality PPC might mitigate PHI privacy concerns among women using a nationally representative survey to influence meaningful policy mandates.

Thus, this study aims to address this gap by utilizing data from the 2011 to 2018 Health Information National Trends Survey (HINTS). This paper seeks to (1) examine the characteristics of women who withhold information from those who don’t, (2) examine trends in women’s health information withholding behaviors, and (3) evaluate the association between PPC and women’s health information withholding behavior adjusting for sociodemographic and health-related factors.

## Methods and materials

### Data source

Data for this study was obtained from the fourth and fifth iterations of the HINTS fielded by the National Cancer Institute. HINTS is a nationally representative sample of noninstitutionalized US adults aged 18 and older that collects health information access and use data. Data for this study comprised respondents from HINTS 4, cycles 1 (2011), 2 (2012), and 3 (2014), and HINTS 5, cycles 1 (2017) and 2 (2018). We restricted our research to the cycles containing questions to our hypothesis of interest.

HINTS 4 employed a mail-based survey approach. The addresses of participants were randomly selected from the US postal services list of residential addresses. Adults within the selected household were chosen to respond to the survey using the next birthday method. The addresses were stratified into three sampling strata to increase precision estimates for minority subpopulations in 2011, 2012, 2014, and 2017 into (1) addresses in areas with high concentrations of minority populations, (2) addresses in areas with low concentrations of minority populations, and (3) addresses located in counties consisting of Central Appalachia irrespective of minority populations. However, only the high-and low-minority strata were used in 2018. The total number of complete responses and the response rates of each survey year in this study were: 2011(3907 and 36.6%), 2012 (3582 and 39.9%), 2014 (3124 and 34.4%), 2017 (3191 and 32.4%), and 2018 (3434 and 32.9%). Detailed information about the HINTS survey methodology is reported elsewhere.

### Measures

#### Outcome variable: information withholding behavior

For our outcome variable, we used the binary (Yes vs. No) response to the HINTS question “Have you ever kept information from your health care provider because you were concerned about the privacy or security of your medical record?” to measure women’s information withholding behaviors.

### Key independent variable

PPC was the key independent variable used for this study. Participants responded to seven separate questions highlighting key aspects of PPC described by Epstein and Street and utilized in several HINTS studies [24, 31]. Specifically, respondents answered on a four-point Likert scale (Always, usually, sometimes, never) on how often they engaged in the following activities with a doctor, nurse, or health professional in the past 12 months: (1) had the chance to ask all the health-related questions they had, (2) received the attention needed for their feelings and emotions, (3) involved them in decisions about their healthcare as much they wanted, (4) made sure they understood the things needed to do to take care of their health, (5) explained things in a way they could understand, (6) spent enough time with them, and (7) helped them deal with uncertainty about their health or healthcare. We recoded the responses so that higher scores reflected positive patient-provider scores (i.e., 1 = never and 4 = always). A PPC composite score was created by summing the individual questions; minimum and maximum scores were 7 and 28, respectively. We achieved a high internal consistency with a Cronbach alpha value (0.93) of the seven PPC items.

### Control variables

Following previous HINTS studies [1, 26, 27], we included sociodemographic and health-related variables as control variables. Sociodemographic characteristics included: age (18–24, 25–34, 35–39, 50–64, or > 65 years); race/ethnicity (non-Hispanic whites, non-Hispanic blacks, Hispanics, Asians, Others, or missing); marital status (single, married/living with a partner, or widowed/divorced/separated); income status (less than $20,000, $20,000 to $34,999, $35,000 - $49,999, $50,000 to $74,999, or $75,000 and above); education status (High school or less, some college degree, or college degree or more); employment status (unemployed vs. employed); residence (non-metropolitan vs. metropolitan). Health-related questions included: having a regular provider (no vs. yes); self-reported health status (fair/poor, good, or very good/excellent); confidence in care (not confident vs. confident); quality of care (good/fair vs. excellent); smoking status (none, former, or current); psychological distress none/minimal, mild, moderate, or severe). The psychological distress score was created by summing and grouping a four-point Likert scale response to an ultra-brief four-validated psychological instrument for assessing depression (PHQ-2 with two items) and anxiety (Generalized Anxiety Disorder-2 with two items) from the patient health questionnaire questionnaire-4 (PHQ-4) used in the literature [24].

### Statistical analyses

Aggregated data from each iteration were weighted to allow population-level inferences using the recommended HINTS analytical strategy: we applied 250 replicate jackknife survey weights (50 jackknife survey weights each year).

The “e-sample” command in STATA 17.0 software was used to balance the sample so that only respondents who answered all the questions across iterations were analyzed. An initial weighted descriptive statistic was conducted using the Chi-square test to compare the characteristics of the study population by withholding behaviors. The weighted mean and standard error (SE) were also computed for discrete measures. Separate multivariate logistic regression models were conducted with the combined years and across each year and adjusted for the control variables. We also graphed the trends of withholding behaviors and assessed the within-group difference between withholding behaviors and the seven PPC items. Missing data or unknown observations were dropped for all analyses except for race/ethnicity. The adjusted odds ratio (aOR), 95% Confidence Interval (CI), and an alpha of < 0.05 for statistical significance were applied. Multicollinearity was investigated using the variance inflation factor, and no collinearity was found among the variables. Hosmer-Lemeshow goodness of fit test was conducted; we found an insignificant chi-square test output, suggesting a good model fit. All analysis was performed with Stata 17.0 SE.

## Results

A total of 10,628 women participated across cycles, of which only 7738 responded to all the measures of interest in this study each year. Overall, 10.8% (95% CI 9.86–11.9%) of women answered that they withheld information from their providers because of privacy or security concerns about their medical records. The mean value and standard error of patient communication score were 23.5 and 0.09 (95% CI; 23.3 to 23.7). A greater share of the sample was non-Hispanic white women (66.2%), married (55.3%), have a regular provider (74.5%), live in a metropolitan area (83.4%), confident about their care (70.6%), and reported an excellent quality of care (76%). Women with information-withholding behaviors had a slightly lower mean patient communication score than those who did not (mean 21.6 vs. 23.7). There was a within-group significant statistical difference between withholding behaviors and age, race/ethnicity, quality of care, and psychological distress (Ps < 0.0001). Educational status, employment status, confidence in care, and smoking status also predicted women’s ability to withhold information from providers because of privacy or security reasons for their medical records (Table 1).

**Table 1:** Characteristics of participants and stratified by withholding behaviors from providers for privacy and security concerns

Variables	All N = 7738 (%)	Withheld information from the provider because of privacy or security concerns about medical records		p-value
		Yes N, (%)	No N, (%)	
		902, (10.8)	6836 (89.2)	
**Patient-provider communication score**, mean (SE)	23.5 (0.09)	21.6 (0.26)	23.7 (0.10)	
Age				< 0.001
18–24	240 (9.6)	24 (0.6)	216 (9)	
25–34	945 (15.5)	144 (2.0)	801 (13.5)	
35–49	1822 (27)	299 (3.8)	1523 (23.1)	
50–64	2604 (28.8)	310 (3.3)	2294 (25.5)	
> 65	2127 (19.1)	125 (1.0)	2002 (18.1)	
**Race/ethnicity**				< 0.001
non-Hispanic whites	4679 (66.2)	462 (6.1)	4217 (60.1)	
non-Hispanic blacks	1221 (11.2)	165 (1.5)	1056 (9.6)	
Hispanic	913 (11.8)	141 (1.6)	772 (10.2)	
Asian	236 (3.4)	47 (0.6)	189 (2.7)	
Others ^a^	285 (2.9)	43 (0.4)	242 (2.5)	
Missing	404 (4.3)	44 (0.3)	360 (3.9)	
**Marital status**				0.788
Single	1279 (25.1)	176 (2.8)	1103 (22.3)	
Married	3821 (55.3)	421 (5.9)	3400 (49.3)	
Widowed/divorced/separated	2638 (19.6)	305 (2.0)	2333 (17.6)	
**Income status**				0.485
Less than $20,000	1663 (19.8)	229 (2.4)	1434 (17.4)	
$20,000 to $34,999	1165 (13.6)	127 (1.5)	1038 (12.1)	
$35,000 to $49,999	1080 (13.8)	120 (1.5)	960 (12.2)	
$50,000 to $74,999	1323 (17.1)	156 (1.8)	1167 (15.3)	
$75,000 and above	2507 (35.6)	270 (3.4)	2237 (32.2)	
**Educational status**				0.049
High school or less	2058 (28.5)	170 (2.5)	1888 (26)	
Some college degree	2295 (35.7)	302 (4.1)	1993 (31.6)	
College degree or more	3385 (35.7)	430 (4.1)	2955 (31.6)	
**Regular provider**				0.247
No	1767 (25.5)	234 (3.0)	1533 (22.5)	
Yes	5971 (74.5)	668 (7.8)	5303 (66.7)	
**Employment status**				0.010
Unemployed	3736 (46.3)	366 (4.3)	3370 (42)	
Employed	4002 (53.7)	536 (6.6)	3466 (47.1)	
**Self-reported health status**				0.145
Fair/poor	1276 (15.3)	186 (1.9)	1090 (13.3)	
Good	2790 (35.5)	312 (3.8)	2478 (31.6)	
Very good/excellent	3672 (49.2)	404 (5.0)	3268 (44.2)	
**Residence**				0.354
Nonmetropolitan area	1161 (16.6)	115 (1.6)	1046 (15)	
Metropolitan area	6577 (83.4)	787 (9.2)	5790 (74.2)	
**Confidence in care**				0.007
Not confident	2267 (29.4)	343 (3.9)	1924 (25.5)	
Confident	5471 (70.6)	559 (6.8)	4912 (63.7)	
**Quality of care**				< 0.001
Good/fair	1851 (24)	327 (3.8)	1524 (20.1)	
Excellent	5887 (76)	575 (6.9)	5312 (69.1)	
**Psychological distress**				< 0.001
None-minimal	5243 (66)	473 (5.9)	4770 (60.1)	
Mild	1482 (19.4)	200 (2.3)	1282 (17.1)	
Moderate	567 (8.0)	114 (1.3)	453 (6.6)	
Severe	446 (6.6)	115 (1.1)	331 (5.3)	
**Smoking status**				0.001
Never	4880 (64.5)	529 (6.0)	4351 (58.5)	
Former	1848 (21.8)	214 (2.7)	1634 (19.1)	
Current	1010 (13.7)	159 (2.1)	851 (11.6)	

a; American Indians/Native Americans

**Fig. 2:**
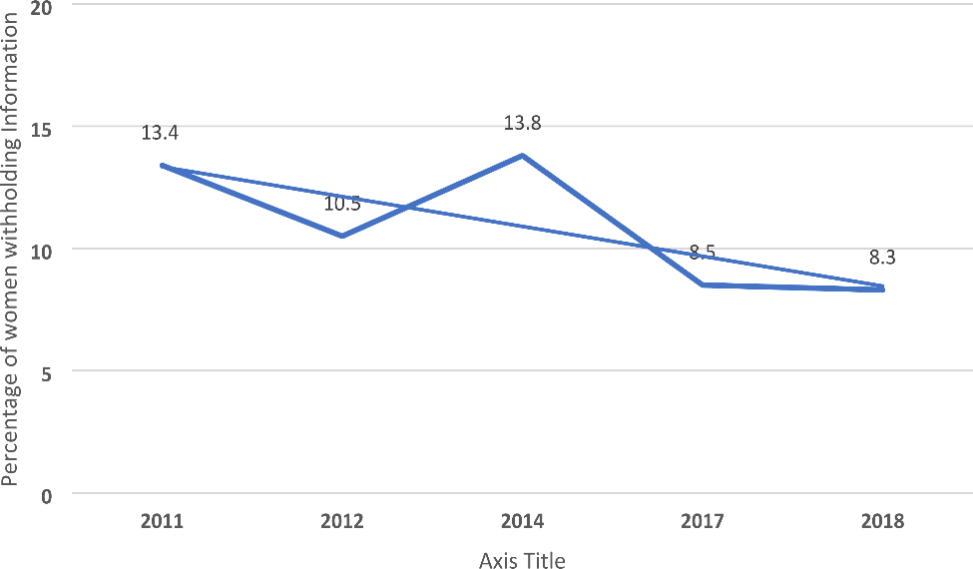
Temporal changes in women’s withholding behavior due to privacy and security concerns in the United States, 2011–2018. *P < 0.001

**Fig. 3:**
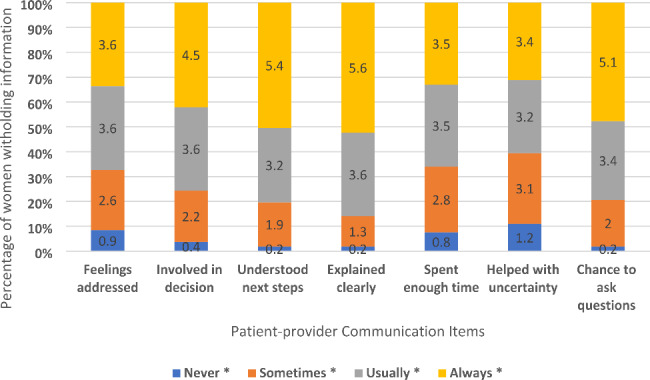
Trends of withholding health information from providers for privacy and security concerns by the seven patient-provider communication items: 2011–2018 among women in the United States

Figure 2 shows a decline in withholding behaviors from 13.4% to 2011 to 8.3% in 2018. Further, as seen in Fig. 3, there was a significant statistical difference between information-withholding behaviors and the seven patient communication items, with the majority of the sample reporting “always and usually vs. never” engaging in communication activities, indicating a positive provider interaction/encounter.

After adjusting for the control variables in the combined dataset (Table 2), a higher PPC score was associated with lower odds of withholding information from the provider because of privacy and security concerns (AOR 0.93, 95% CI: 0.90–0.95, P < 0.001). The same pattern was found across each year after controlling for variables: 2011(AOR 0.94, 95% CI: 0.90–0.99, P = 0.045), 2012 (AOR 0.97, 95% CI: 0.90–1.04, P = 0.475), 2014 (AOR 0.91, 95% CI: 0.85–971, P = 0.005), 2017 (AOR 0.92, 95% CI: 0.85–0.99, P = 0.043), and 2018 (AOR 0.87, 95% CI: 0.81–0.93, P < 0.001). However, in 2012, no statistically significant relationship was found between PPC and withholding behaviors, Table 3.

Concerning the control variables, age, race/ethnicity, educational status, psychological distress, and smoking status significantly predicted women’s willingness to disclose health information in the combined dataset (Table 2). However, across years (full model of Table 3 not shown), in 2011, age, income levels, educational status, employment status, and smoking status were associated with the willingness to share information. In 2012, only age and psychological distress predicted information disclosure. Marital status and psychological distress were predictors of disclosing health information in 2014. Only marital status was associated with willingness to disclose health information in 2017. Moreover, in 2018, age, race/ethnicity, and smoking status were significant.

**Table 2:** Association between patient-provider communication and withholding information from providers for privacy and security concerns between 2011–2018 among women in the United States

Variables	All, N = 7738 aOR, 95% CI	p-value
**Patient-provider communication score**	0.93 (0.90–0.95)	< 0.001
**Age**		
18–24	0.88 (0.40–1.95)	0.760
25–34	1.90 (1.23–2.95)	0.004
35–49	2.36 (1.60–3.49)	< 0.001
50–64	2.01 (1.41–2.87)	< 0.001
> 65	Ref	
**Race/ethnicity**		
non-Hispanic whites	Ref	
non-Hispanic blacks	1.52 (1.05–2.20)	0.024
Hispanic	1.73(1.25–2.40)	0.001
Asian	2.32(1.41–3.79)	0.001
Others ^a^	2.06 (1.11–3.80)	0.021
Missing	1.19 (0.75–1.87)	0.446
**Marital status**		
Single	Ref	
Married	1.03 (0.74–1.42)	0.847
Widowed/divorced/separated	1.08 (0.78–1.51)	0.615
**Income status**		
Less than $20,000	Ref	
$20,000 to $34,999	0.93 (0.58–1.48)	0.769
$35,000 - $49,999	0.89 (0.58–1.38)	0.627
$50,000 - $74,999	0.86 (0.56–1.30)	0.483
$75,000 and above	0.72 (0.48–1.08)	0.114
**Educational status**		
High school or less	Ref	
Some college degree	1.45 (1.06-2.00)	0.020
College degree or more	1.45 (1.03–2.04)	0.032
**Regular provider**		
No	Ref	
Yes	1.11 (0.86–1.44)	0.381
**Employment status**		
Unemployed	Ref	
Employed	1.14 (0.84–1.54)	0.384
**Self-reported health status**		
Fair/poor	Ref	
Good	1.09 (0.76–1.57)	0.627
Very good/excellent	1.42 (0.96–2.11)	0.076
**Residence**		
Nonmetropolitan area	Ref	
Metropolitan area	1.04 (0.74–1.46)	0.807
**Confidence in care**		
Not confident	Ref	
Confident	0.88 (0.68–1.14)	0.356
**Quality of care**		
Good/fair	Ref	
Excellent	0.89 (0.66–1.19)	0.445
**Psychological distress**		
None-minimal	Ref	
Mild	1.24 (0.92–1.67)	0.148
Moderate	2.04 (1.42–2.92)	< 0.001
Severe	1.93 (1.23–3.02)	0.004
**Smoking status**		
Never	Ref	
Former	1.61 (1.22–2.12)	0.001
Current	1.59 (1.15–2.21)	0.005
**Year**		
2011	Ref	
2012	0.69 (0.48-1.00)	0.051
2014	1.06 (0.78–1.44)	0.691
2017	0.57 (0.47–0.82)	0.002
2018	0.59 (0.40–0.88)	0.011

a; American Indians/Native Americans

**Table 3:** Association between patient-provider communication and withholding information from providers for privacy and security concerns across years

Variables	2011, N = 1660 AOR, 95% CI	2012, N = 1532 AOR, 95% CI	2014, N = 1579 AOR, 95% CI	2017, N = 1423 AOR, 95% CI	2018, N = 1544 AOR, 95% CI
Patient-provider communication score	0.94 (0.90–0.99) P = 0.045	0.97 (0.90–1.04) P = 0.475	0.91 (0.85–971) P = 0.005	0.92 (0.85–0.99) P = 0.043	0.87 (0.81–0.93) P < 0.001

All models adjusted for all covariates included in this study: Age, Race/ethnicity, Marital status, Income status, Educational status, Regular provider, Self-reported Health status, Residence, Confidence in care, Quality of care, Psychological distress, Smoking status

## Discussion

This study investigated whether PPC impacts women’s willingness to disclose health information to their providers due to security concerns about their medical records. Although there was a decline in withholding information from 13.4 to 8.3% between 2011 and 2018, an estimated 1 in 10 women in the US did not disclose health information within the same period. After adjusting for the control variables, an increase in PPC scores was significantly associated with a decreased odds of nondisclosure of health information for security concerns by 7% and ranged between 9% and 13% each year. Our findings highlight the need to adopt multilevel strategies, using an ecological perspective to improve quality patient-provider relationships and interactions to protect and promote women’s health in the US.

Concerning the sociodemographic characteristics, we found that age (between 25 and 64 years vs. 65 and older), race/ethnicity (black, Hispanic, Asian, and tribal women vs. white women), and educational status (having some or a college degree and above vs. high school or less) increased the likelihood of withholding health information from their providers for security reasons. This corroborates the literature, demonstrating that certain subgroups of patients are more predisposed to such behaviors [1, 26]. In light of the increasing health disparities in the US and the importance of EMRs, strategies must be adopted to mitigate women’s concerns about disclosing critical health information to providers for optimal health outcomes. For example, women’s health needs vary across age groups, which calls for opportunities for sex-and gender-inclusive care to prevent stereotypes during medical consultation, enhancing health communication [30]. Furthermore, the revelation that ethnic/minority women tend to withhold health information is sadly not far-fetched. Minoritized women experience racially motivated care, leading to medical distrust and worse health outcomes across several health indices, thus calling for culturally concordant care [11]. On the other hand, women with higher levels of education may have higher literacy and structural advantage of seeking information from alternative health sources, such as the internet [32], instead of disclosing PHIs if they perceive they are receiving poor quality health services. They may also have private/comprehensive health insurance to improve their access to quality health services [33].

Consistent with previous studies [6, 26], we found that smoking status and poor mental health were associated with the nondisclosure of health information. While previous studies generally use aggregated data (i.e., controlling for sex), which might lead to biased estimates concerning the true effect of women’s nondisclosure behaviors, disaggregated data increases measurement validity to substantiate our findings. Since smoking and poor mental health adversely affect maternal and child health [34], withholding medical information could delay the diagnosis and treatment of mental disorders and/or co-occurring substance misuse in this vulnerable group. Withholding behaviors may also prevent admittance and timely access to public health prevention programs such as smoking cessation. Findings affirm the need for strategies to address the stigmatization of smoking, poor mental health, or fear of health insurance penalties that hinder willingness to disclose pertinent health information.

As mentioned earlier, quality PPC is implicated in promoting equitable health outcomes. While there are numerous reasons why women do not disclose pertinent health information, it is relevant that non-medical influences, particularly given the current US sociopolitical landscape, that substantiate nondisclosure behaviors are recognized and addressed systematically [35]. For example, during medical consultations, providers could educate women about the potential risks associated with online generated data, considering most women’s mHealth apps have suboptimal data privacy, sharing, and security standards that do not meet regulatory guidelines [13]. In fact, 87% of 23 mHealth apps shared user data with third parties, yet only 52% requested explicit user consent [13]. Such advice has consequences across all aspects of women’s health. Providers could also reduce, where probable, the amount of data collected to prevent the data from being maliciously used [7, 36].

Furthermore, providers could use their clinical and ethical judgment to consider when an in-person visit may trump and supplant communication via email, text messaging, or EMRs. Even though providers do not require patients’ consent due to mandatory reporting laws when reporting certain medical encounters. However, it would be courteous to notify their patients or consider transformative and restorative interventions such as utilizing community-based processes outside the criminal justice system before and, when necessary, reporting to the appropriate authority [37–39]. Because mandatory laws tend to be more punitive towards minority populations, including women and racial/ethnic groups, such an approach is germane when considering the fact that mandatory reporting laws are associated with reduced odds of help-seeking behaviors from medical providers, shelters, or family and friends among domestic violence survivors [38]. One in three survivors, regardless of gender, race/ethnicity, and sexual orientation, did not seek the needed support for mandatory reporting laws.

Further, mandatory reporting laws increased the fear of criminal investigation, involvement with child protective services, fear of homelessness, fear of deportation, or worsened living conditions, exposing survivors to more danger than before the reporting event [38]. Transformative approaches may foster trust accountability, prevent future harms, and confidence between the patients and providers, improving PPC. Codifying terminologies and languages (if possible and reasonable) when documenting sensitive information in ways that do not connote illegal activity could be considered [7, 36].

Beyond the impact of health laws on PPC, the Ecological Model of Communication in Medical Encounters posits that the media context (e.g., social media) and the organizational context (e.g., health insurance) also influence the PPC process and, ultimately, health outcomes. For example, women with Medicaid, uninsured, and other HMO-based care have disparate care, including poor PPC, compared to those who are privately insured or through an employer [33, 40–42]. Further, despite the substantial benefits of social media on health delivery, serious privacy, and confidentiality concerns can negatively affect women’s health [43, 44]. Although this study did not measure how external factors moderate PPC and women’s privacy concerns, it is evident that there is a need for structural strategies to promote women’s health. The Hospital Consumer Assessment of Healthcare Providers and Systems scores could be expanded to include metrics that measure external influences to strengthen quality improvement strategies, including PPC quality [45]. Expansions to Medicaid [46] to protect perinatal health should still be considered. Moreover, future research should assess how PPC moderates women’s privacy concerns using ecological, longitudinal, and qualitative measures to promote and protect women’s health holistically.

### Implications for public health research and policy

Considering the breadth of women’s health, withholding medical information has serious implications not only for the women themselves but also society. Witholding medical information could compromise the health of women and their families, healthcare surveillance systems, and society. Women, particularly minority women, must practice self-advocacy by researching the applicable hospital, state, and federal laws to make an informed decision about their care when selecting providers, health insurers, or other stakeholders that promote women’s privacy rights. Being actively engaged during medical consultations by asking questions or seeking clarifications about the benefits-risks of sharing sensitive health information could also empower health-seeking behaviors. Health systems across the continuum of care must proactively educate their providers and staff on health laws and how to effectively deliver quality healthcare services despite the health landscape. They must also implement strong internal infrastructure to curtail medical breaches or other malpractices while also adopting system-level changes such as collaborative model of care involving a community-based process to decriminalize certain health behaviors Providers and health systems must reorient themselves towards any gender/racially-based stereotypes or socially construed biases that undermine the quality of care they deliver to women. Further, policymakers must continue to enhance and support sex-and gender-inclusive policies to amplify and promote women’s health. Due to the limitations of HIPAA, several stakeholders have heralded the reformation of these laws to protect vulnerable populations better and from unwarranted patient-provider relationship intrusion. Therefore, it is imperative that policymakers and health systems to provide an abridged version of HIPAA to provide layperson education on the law for patients.

### Study’s strengths and limitations

To the best of our knowledge, this is the first study to investigate the relationship between PPC and nondisclosure behaviors among women using nationally representative data. However, this study has some limitations. First, this study utilizes survey data prone to selection and recall bias for some measures, although applying the jackknife survey weights may reduce the bias. Second, we cannot confirm which health information women withheld because HINTS did not collect such data. Even though women have more privacy concerns than men, it may be that these concerns are fueled by health conditions (e.g., reproductive care or psychosocial health) or unrelated to a health condition (e.g., communication barriers). Third, we cannot explicitly infer causal inference as with other cross-sectional studies. Another noteworthy limitation is that we did not measure potential confounders that may influence PPC since they were not captured in the HINTS dataset. For example, it is plausible that sociodemogra characteristics such as gender, age and race/ethnicity (especially given the noted significant impact of race and ethnicity in bivariate analyses) may affect the patient-provider dynamic. Other unmeasured cofounders that may have significant impact on PPC and withholding behavior are provider experience and expertise, trust, and stigma (e.g., the bivariate relationship between the stigmatized behavior of smoking and information withholding). Despite these limitations, this study provides a blueprint for future research to investigate how PPC can protect and promote women’s health in the US.

## Conclusions

Provider relationship with patients is integral to optimal health outcomes and patient experience. Our study suggests that improving PPC quality may reduce women’s privacy and security concerns, encouraging them to disclose sensitive medical information. This underscores the need for providers to build trust and empathy to buffer privacy and security concerns in women.

## Data Availability

All data generated and analyzed in this study can also be requested freely via https://hints.cancer.gov/.

## Abbreviations

EMR: Electronic medical record
PHI: Protected health information
US: United States
HIPAA: Health Insurance Portability and Accountability Act
PPC: Patient-provider communication
HINTS: Health Information National Trends Survey
SE: Standard error
AOR: Adjusted odds ratio
CI: Confidence Interval
